# Prospective Multicenter Open-Label One-Arm Trial Investigating a Pumpkin Seed, Isoflavonoids, and Cranberry Mix in Lower Urinary Tract Symptoms/Benign Prostatic Hyperplasia: A Pilot Study

**DOI:** 10.1155/2020/6325490

**Published:** 2020-01-13

**Authors:** Elie Nemr, Elie El Helou, Georges Mjaess, Albert Semaan, Josselin Abi Chebel

**Affiliations:** Université Saint Joseph, Faculty of Medicine, Beirut, Lebanon

## Abstract

Phytotherapy for lower urinary tract symptoms (LUTSs) due to benign prostate hyperplasia (BPH) is progressively demanded by patients and trusted by physicians. The aim was to assess the efficacy of a mix of pumpkin seed extract, soy germ isoflavonoids, and cranberry (Novex®) in the management of mild to moderate LUTS in BPH patients. Male patients aged ≥40 years, who had had mild to moderate LUTS for >6 months at screening, with no previous therapy or who are still symptomatic despite current use of alpha-blockers, were recruited. Exclusion criteria were an IPSS >19 and an age >80 years. The mixed compound was administered orally, daily, for 3 months. Patients were evaluated by means of IPSS, urological quality of life (uQoL) index, and International Index of Erectile Function (IIEF-5) at 3 visits: baseline (visit 1), 30 days (visit 2), and 90 days after treatment (visit 3). Among 163 screened patients, 128 patients (61.8 ± 9.9 years) were recruited. IPSS improved from 15 (Q1 : 12–Q3 : 17) in visit 1, to 11 (Q1 : 8–Q3 : 14) in visit 2, and to 9 (Q1 : 6–Q3 : 12) in visit 3 (*p* < 0.001). uQoL improved from 4 (3–4) in visit 1, to 3 (2–3) in visit 2, and to 2 (1–2) in visit 3 (*p* < 0.001). The patients had an IIEF-5 score of 15 (12–18.7) in visit 1, 15 (12–18) in visit 2, and 17 (13–19) in visit 3 (*p*=0.99 visits 1 vs. 2, *p*=0.004 visits 2 vs. 3, and *p*=0.001 visits 1 vs. 3). Treating mild to moderate LUTS/BPH patients with Novex® might therefore relieve symptoms, improve the quality of life, and have a mild beneficial effect on erectile function.

## 1. Introduction

Phytotherapy for lower urinary tract symptoms (LUTSs) due to benign prostate hyperplasia (BPH) is being increasingly demanded by patients and trusted by physicians, reaching roughly 90% in some countries like Germany and Austria [1]. Phytotherapy is mainly used for mild to moderate LUTS while guidelines still advocate the use of alpha-blockers and phosphodiesterase 5 inhibitors for moderate to severe LUTS [2, 3].

Among many phytotherapeutic compounds available and studied in the literature, pumpkin seed extract, soy germ isoflavonoids, and cranberry have shown efficacy and safety in the treatment of LUTS. Pumpkin seed is the most studied nutraceutical and has shown a significant improvement in LUTS and in urological quality of life. Fewer studies evaluated the role of soy germ isoflavonoids and cranberry and showed a beneficial effect on LUTS.

BPH patients presenting with LUTS have a high rate of erectile dysfunction (ED) and LUTS is considered as an independent risk factor for ED [4, 5]. Treatment of LUTS with alpha-blockers has been associated with improvement of erectile function [6], but the effect of the nutraceuticals used to treat LUTS on erectile function remains elusive. To date, no studies have assessed the effect of a combination of the three compounds (pumpkin seed extract, soy germ isoflavonoids, and cranberry) on LUTS in BPH patients and their impact on the urological quality of life.

Therefore, the aim of the current study is to assess the efficacy and safety of a new complex containing pumpkin seed extract, soy germ isoflavonoids, and cranberry in the treatment of mild to moderate LUTS and erectile dysfunction in LUTS/BPH patients.

## 2. Materials and Methods

### 2.1. Study Design and Setting

This is a single-arm prospective open-label trial investigating a combination of pumpkin seed, isoflavonoids, and cranberry mix in improving LUTS, urological quality of life, and erectile function in BPH patients. The study took place in outpatient settings in several clinics across Lebanon. An Institutional Review Board approval was obtained, and patients signed a written informed consent form. The urology specialists in the outpatient clinics were introduced to the study protocol by the investigators and were invited to recruit the patients who satisfied the eligibility criteria. Recruitment started in April 2018 and lasted until October 2018 (last patient last visit). Recruited patients were seen at 3 visits: initial visit (visit 1), 1-month visit (visit 2), and 3-month visit (visit 3), and their data were collected in a dedicated case report form. The compound was administered orally, two tablets daily, for 3 months.

### 2.2. Investigational Drug

**Figure 1:**
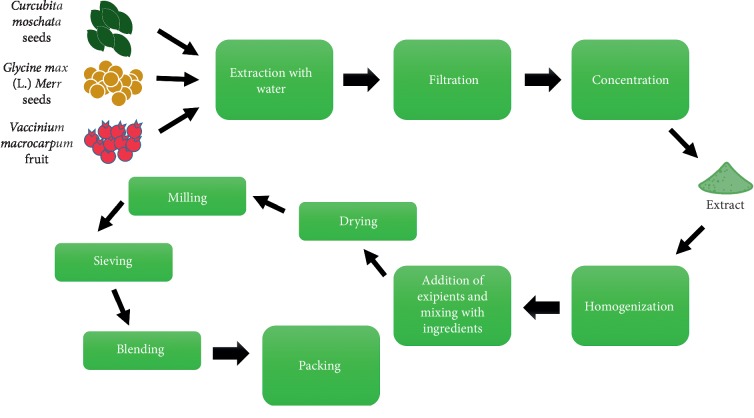
Process flowcharts of pumpkin seed extract, soy extract 40% isoflavones, and cranberry.

Novex® is a nutraceutical complex made of pumpkin seed extract 550 mg, soy germ isoflavonoids 50 mg, and cranberry 50 mg. The recommended dose is 2 tablets/day taken orally. Pumpkin seed, which is the main component in this complex, was produced through a proprietary PS990 HyperPure process that ensures highly selective removal of the fat-soluble components. The flowchart of the production process of each component is shown in Figure 1.

### 2.3. Participants

Male subjects seen at the outpatient clinics between January and October 2018 were eligible to participate in the study if they fulfilled the following criteria: (1) age ≥40 years; (2) with mild to moderate LUTS for at least 6 months at the initial visit; (3) and who have no previous therapy or are still symptomatic despite the current use of alpha-blockers. Exclusion criteria were as follows: (1) an International Prostate Symptom Score (IPSS) > 19, (2) an age >80 years, (3) prostate cancer, and (4) urethral stenosis. Enrolled patients were invited to an assessment visit at the 1-month and the 3-month visits.

### 2.4. Variables

The patients were evaluated using detailed urological history and a clinical examination, and the outcome was measured using the following validated scales:

- IPSS: International Prostate Symptom Score, with its subscores for voiding, storage, and nocturia
- Quality of life due to urinary symptoms (uQoL) index
- International Index of Erectile Function (IIEF-5)

The scales were used at baseline (visit 1) to derive baseline values for IPSS, uQoL, and IIEF-5. The scales were reused at 1-month (visit 2) and 3-month visit (visit 3) to derive follow-up values of the same score in a repeated-measures design. The data were recorded by the specialist during the scheduled visit.

The primary outcome was any change in the IPSS at 1- and 3-month visits. Secondary outcomes were any change in IPSS subscores, uQoL, IIEF-5, and the rate of ejaculatory dysfunction and orthostatic hypotension.

### 2.5. Study Size

Basing the calculations on *F* tests for within-factors ANOVA with repeated measures, the following assumptions were taken conservatively: effect size *f* = 0.20; error probability *α* = 0.05; power (1-*β* error probability) = 0.90; number of groups = 1; number of measurements = 3; correlation among repeated measures = 0.3; and nonsphericity correction *ε* = 0.5. The total sample size satisfying these assumptions is 125 subjects.

### 2.6. Statistical Methods

The distribution of IPSS score (in addition to voiding, storage, and nocturia IPSS subscores), IIEF-5 score, QoL, and optional tests was checked for departure from normality assumptions using Kolmogorov–Smirnov test and visual inspection by quantile-quantile plots. The scores were expressed as median with its interquartile range (Q1–Q3). To take into account the repeated-measures design, for IPSS, uQoL, and IIEF-5 at the initial visit, 1-month visit, and 3-month visit, Friedman's nonparametric repeated-measures ANOVA by ranks was used. Post hoc pairwise comparisons were used, and *p* values were adjusted for multiplicity. Subscores of IPSS for nocturia, filling, and voiding were also analyzed in a similar fashion. Analyses were run using SPSS software (IBM Corp. Released 2013, SPSS Statistics for Windows version 22.0, Armonk, NY).

## 3. Results and Discussion

### 3.1. Patient Characteristics

163 patients were screened, of whom 140 patients were enrolled in the study and 12 patients dropped out. The final number of patients included in the analysis was 128, aged 61.8 ± 9.9 years.

### 3.2. Main Results

**Figure 2:**
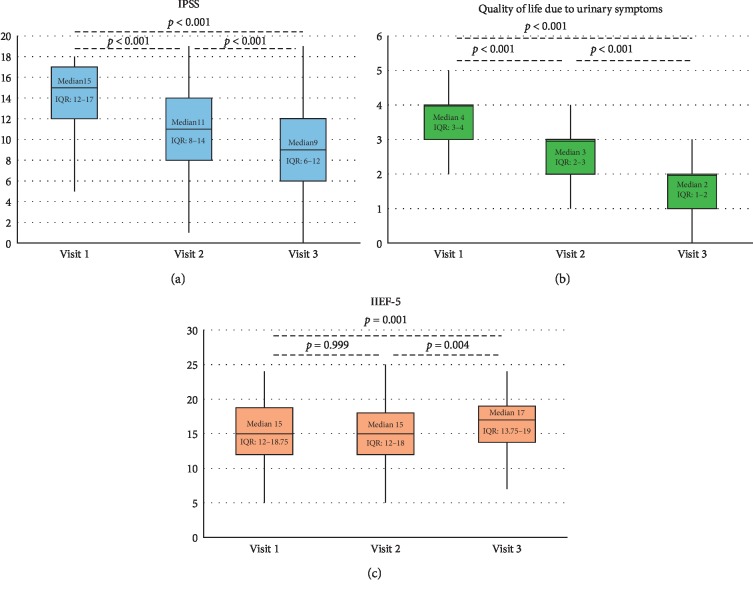
(a) International Prostate Symptoms Score (IPSS), (b) urological quality of life (uQoL), and (c) International Index of Erectile Function (IIEF-5) obtained at the three visits: median, Q1, Q3, minimum, and maximum.

Patients had a baseline median IPSS score of 15 (12–17), significantly decreasing to 11 (8–14) at 1-month visit and then to 9 (6–12) at 3-month visit (*p* < 0.001). Post hoc analysis using pairwise comparisons showed a significant difference between visit 1 and visit 2 (*p* < 0.001), visit 2 and visit 3 (*p* < 0.001), and visit 1 and visit 3 (*p* < 0.001) (Figure 2(a)).

**Figure 3:**
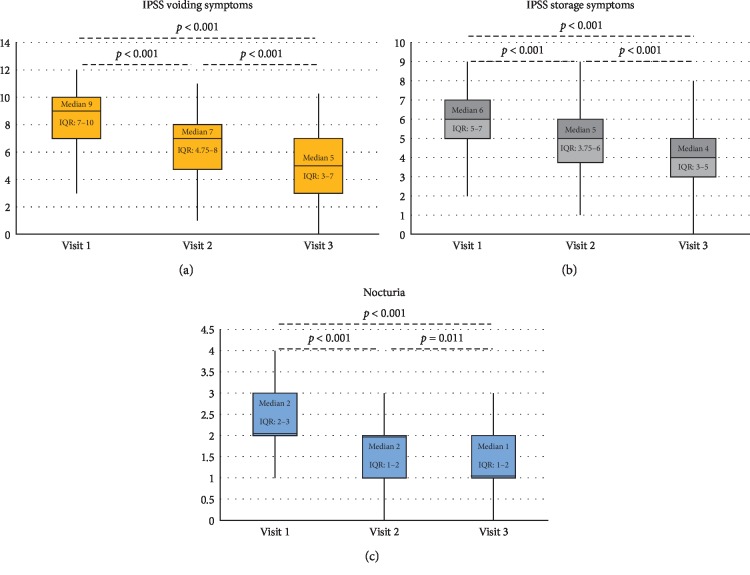
(a) IPSS voiding, (b) IPSS storage, and (c) nocturia subscores obtained at the three visits: median, Q1, Q3, minimum, and maximum.

As for the secondary outcomes, the same pattern of variation was observed when analyzing voiding, storage, and nocturia IPSS subscores. Median IPSS voiding symptom score significantly decreased from baseline 9 (7–10) to 7 (4.75–8) at 1-month visit and then to 5 (3–7) at 3-month visit (*p* < 0.001) (Figure 3(a)). Median IPSS storage symptoms score significantly decreased from baseline 6 (5–7) to 5 (3.75–6) at 1-month visit and then to 4 (3–5) at 3-month visit (*p* < 0.001) (Figure 3(b)). IPSS nocturia score significantly decreased from baseline 2 (2–3) to 2 (1–2) at 1-month visit and then to 1 (1–2) at 3-month visit (*p* < 0.001) (Figure 3(c)). Post hoc analyses of the subscores showed a significant difference between visit 1 and visit 2 (*p* < 0.001), visit 2 and visit 3 (*p* < 0.001), and visit 1 and visit 3 (*p* < 0.001 for voiding and storage symptoms, *p*=0.011 for nocturia).

Regarding the quality of life related to urological symptoms (uQoL), 39.1% of the patients were mostly dissatisfied and 14.1% were unhappy, making a total of 53.2% having a low quality of life at baseline visit. At 1-month visit, 15.6% of the patients were mostly dissatisfied and 2.3% were unhappy, making a total of 17.9% having a low quality of life. At 3-month visit, 7.0% of the patients were mostly dissatisfied and 3.9% were unhappy, making a total of 10.9% having a low quality of life. uQoL significantly decreased from a median of 4 (3–4) at baseline to 3 (2–3) at 1-month visit and then to 2 (1–2) at 3-month visit (overall *p* < 0.001). The post hoc analyses showed that this difference also stemmed from the difference between all 3 visits (all *p* values <0.001) (Figure 2(b)).

At baseline, the International Index of Erectile Function (IIEF-5) score was 15 (12–18.75); it remained at a median of 15 (12–18) at 1-month visit and then increased to 17 (13.75–19) at 3-month visit (overall *p* < 0.001). The post hoc analysis showed no significant difference between visit 1 and visit 2, but the difference between visit 2 and visit 3 was significant (*p*=0.004) (Figure 2(c)).

Neither ejaculatory dysfunction nor orthostatic hypotension was reported by the patients (0%).

### 3.3. Discussion

The use of nutraceuticals as phytotherapy for the relief of LUTS due to BPH seems to be gaining momentum worldwide and is being considered by clinicians as a treatment option in the management of mild to moderate LUTS/BPH patients. On the other hand, urological societies scarcely mention phytotherapy and do not recommend until now the use of phytotherapeutic agents due to the heterogeneity of their composition and clinical effects, as well as the lack of evidence regarding their benefit [2, 3].

The current study assessed the efficacy of a nutraceutical complex made of pumpkin seed extract, soy germ isoflavonoids, and cranberry, in the management of male patients suffering from mild to moderate LUTS/BPH. The results show that (1) the IPSS score decreased significantly at one and three months of treatment (for the voiding, storage, and nocturia subscores); (2) the uQoL score improved significantly after one and three months of treatment, and a significant inferior proportion of low quality of life was found among patients after one and three months of treatment; and (3) it took three months for the IIEF-5 score to improve significantly.

Several studies performed in the preclinical and clinical setting showed that the use of *Cucurbita pepo* (pumpkin seeds) in the management of patients affected by LUTS/BPH seems to be useful for improving symptoms and quality of life. In the preclinical setting, pumpkin seed showed an antioxidant, antiandrogen, and anti-inflammatory activity and had a positive effect on bladder contractility and reduction of prostate gland growth, while not having any side effects [7]. Tsai et al. showed that the use of pumpkin seed decreases the size of the prostate in testosterone/prazosin-induced prostatic growth [8]. Hong et al. showed, in a placebo-controlled study, a decrease of the IPSS score and an improvement in the urologic quality of life, in patients treated with pumpkin seed extract [9]. Vahlensieck et al. showed that treatment with pumpkin seed results in a significant improvement in BPH/LUTS and a clinically significant improvement in IPSS-related QoL [10].

The soy germ isoflavones showed good effect in treating BPH in men, via their antioxidative properties. In the preclinical setting, soybean isoflavones have been shown to decrease PSA expression when cultured with prostate cancer cells [11]. A significant reduction of PSA has been also reported using an isoflavone-rich supplement as part of an intensive diet and lifestyle intervention [12], and trends toward a reduction in PSA have been noted from some other small trials [13]. Bae et al. reported an amelioration of the IPSS score, storage symptoms, and quality of life in men taking Seoritae extract, a type of black soybean containing isoflavone, at 4 and 12 weeks of administration [14]. Wong et al. showed amelioration in *Q*_max_ and incomplete emptying in patients taking 40 mg of isoflavones daily compared to placebo [1]. Both studies showed excellent tolerability of isoflavones and no particular side effects.

Although cranberry has been evaluated thoroughly in recurrent urinary tract infection prophylaxis [15], its role in the treatment of LUTS in males with BPH has also been evaluated. The first trial evaluating cranberry in the treatment of LUTS, elevated PSA levels, and nonbacterial prostatitis was conducted by Vidlar et al. in 2010 [16]. In this trial, 42 patients were randomized equally to 1500 mg of cranberry fruit powder (*Vaccinium macrocarpon*) daily versus placebo. Six months later, patients who received cranberry had a statistically significant lower IPSS and QoL score than controls. All parameters of urination (*Q*_max_, average urinary flow rate (*Q*_ave_), and prostate bladder voiding and postvoid residual urine volumes) were significantly improved. Furthermore, in a 6-month, randomized, double-blind, placebo-controlled study, Vidlar et al. evaluated cranberry powder (Flowens™) 250 mg and 500 mg versus placebo showing a clinically relevant, dose-dependent, and significant reduction in LUTS in moderately symptomatic men over 45 [17].

Since each compound has been shown to act through a different mechanism of action, giving minor results in the studies abovementioned, the authors think that combining these effects altogether might give an additional clinical value. The combination of these three compounds seems to be promising as shown by this study: the median IPSS decreased by 6 points (15 to 9) after 3 months, which is higher than the predefined 3 points necessary for a patient to perceive a clinical benefit as described in the literature [18]. These results are comparable with those obtained by the use of alpha-blockers in moderate to severe LUTS as reported in the meta-analysis done by Yuan et al. with a decrease of IPSS of 5.4–7 points [19]. This improvement in the IPSS was due to a decrease in voiding and storage subscores. Therefore, this phytotherapy could be beneficial for all mild to moderate LUTS/BPH patients independently of their LUTS type.

Patients with mild LUTS are usually treated with behavioral modifications and watchful waiting until their LUTS becomes moderate (until the benefit of medical treatment outweighs the side effects). Having no adverse events, Novex® may be a suitable choice for mild LUTS/BPH patients. Furthermore, it might be offered as a suitable treatment for patients with moderate LUTS, alone, or in combination with alpha-blockers, having an efficacy comparable to alpha-blockers in this category of patients. The role of nutraceuticals in the treatment of severe LUTS/BPH alone or as an add-on for alpha-blockers needs to be assessed in further studies.

This study is considered a pilot study and has limitations. First, it is a noncomparative study lacking a control arm and using a subjective outcome measure based on the patients' impression. Second, a follow-up of 3 months is relatively short. Therefore, a prospective controlled study with a longer follow-up, and using objective outcome measures (uroflowmetry, voiding diary, etc.), comparing the effect of this phytotherapeutic compound to a placebo group, is being planned.

## 4. Conclusions

This pilot study seems promising, demonstrating that an oral combination of pumpkin seed extract, soy germ isoflavonoids, and cranberry could be beneficial for BPH patients with mild to moderate LUTS, and to our knowledge, this is the first study to assess the beneficial effect of phytotherapy used primarily for LUTS/BPH patients on erectile function, which seems to take more time to occur when compared to the fast amelioration of LUTS. Future randomized placebo-controlled trials should be done in order to prove its efficacy alone or in combination with an alpha-blocker to improve urinary and sexual function.

## Data Availability

The data used to support the findings of this study are available from the corresponding author upon request.
